# Wing Kinematics-Based Flight Control Strategy in Insect-Inspired Flight Systems: Deep Reinforcement Learning Gives Solutions and Inspires Controller Design in Flapping MAVs

**DOI:** 10.3390/biomimetics8030295

**Published:** 2023-07-07

**Authors:** Yujing Xue, Xuefei Cai, Ru Xu, Hao Liu

**Affiliations:** 1 Shanghai Jiao Tong University and Chiba University International Cooperative Research Center (SJTU-CU ICRC), 800 Dongchuan Road, Minhang District, Shanghai 200240, China; xueyujing@chiba-u.jp (Y.X.); caixuefei@alumni.sjtu.edu.cn (X.C.); chibaxuru@gmail.com (R.X.); 2 Graduate School of Engineering, Chiba University, 1-33 Yayoi-cho, Inage-ku, Chiba 263-8522, Japan

**Keywords:** insect flight, stabilization control, deep reinforcement learning, flight dynamics, unsteady aerodynamics

## Abstract

Flying insects exhibit outperforming stability and control via continuous wing flapping even under severe disturbances in various conditions of wind gust and turbulence. While conventional linear proportional derivative (PD)-based controllers are widely employed in insect-inspired flight systems, they usually fail to deal with large perturbation conditions in terms of the 6-DoF nonlinear control strategy. Here we propose a novel wing kinematics-based controller, which is optimized based on deep reinforcement learning (DRL) to stabilize bumblebee hovering under large perturbations. A high-fidelity Open AI Gym environment is established through coupling a CFD data-driven aerodynamic model and a 6-DoF flight dynamic model. The control policy with an action space of 4 is optimized using the off-policy Soft Actor–Critic (SAC) algorithm with automating entropy adjustment, which is verified to be of feasibility and robustness to achieve fast stabilization of the bumblebee hovering flight under full 6-DoF large disturbances. The 6-DoF wing kinematics-based DRL control strategy may provide an efficient autonomous controller design for bioinspired flapping-wing micro air vehicles.

## 1. Introduction

Flapping insects enable outperforming stability and maneuverability under a wide array of disturbances such as wind gusts and turbulence. Although the small insect body is susceptible even to gentle disturbance, flapping-wing insects are able to restore large deviations through continuous adjustments on wing kinematics within several wing-beat strokes [1,2,3]. The insect flight control system is a highly integrated, closed-loop system [4], in which the nonlinear dynamic system couples the motion equations for body dynamics and the Navier–Stokes equations for unsteady aerodynamics [5]. The nonlinear control strategy required for insect flight stabilization in case of large perturbations in full degrees of freedom is still limited for controller design.

Based on the assumption of rigid flapping-wing aerodynamics, the instability in hovering flight is reported to exist in most flying insects [6,7,8]. With the mechanical sensory and vision systems, the translational (forward/backward, lateral, and vertical) and rotational (roll, pitch, and yaw) deviations of an insect’s body under disturbances can be detected, and further actively corrected via wing kinematics modulations with low latency. Previous studies focused on linear control theory demonstrate the feasibility of proportional derivative (PD) strategy for insect flight control. The linear PD controller is suggested to be an efficient tool for 1-degree-of-freedom (DoF) control [2,3,9,10] in various insects’ flights, as well as 3-DoF control for longitudinal motions [11] and body attitudes under small perturbations of 189.5°/s [12] in bumblebee hovering flight. It is also reported to be effective in full 6-DoF hovering control of bumblebee flight under both small (0.03 m/s) and large perturbations [13], in which the adjustment on control parameters of proportional and derivative gains can be obtained based on a CFD data-driven aerodynamic model (CDAM) and a simplified flight dynamic model. However, several limitations still exist for the traditional control strategies. Firstly, the longitudinal and lateral equations tend to be decoupled and resolved to achieve the longitudinal and sideways control separately [14,15], which leaves the coupling features among six degrees of freedom under large disturbances to be neglected. Moreover, the linear assumption with cycle-averaged model may not hold for some large perturbations, as the nonlinearity exists in the correlations between the wing kinematics modulation and the production of aerodynamic forces and torques [14,16]. More importantly, the precise control parameters were determined through the eigenvalue and eigenvector analyses [12,14], and even optimized using the Laplace transformation and root locus approach [11,13]. This requires time-consuming experiments for optimal parameter achievement as well as the prescribed implementation into the flight system before each task. Considering the existing limitations, a more feasible option for a bioinspired intelligent controller designed for large disturbance conditions based on autonomous a deep reinforcement learning algorithm needs to be further explored.

Flying animals tend to develop their control skills via a trial-and-error evolutionary process, which is consistent with the reinforcement learning (RL) [17] process to work out which behavior interacting with the environment will maximize the rewards. Due to the nonlinear motions and continuous action–state spaces for biomimetic aerial vehicles, the deep reinforcement learning (DRL) controller is proven to give solutions for severe disturbance conditions and complex maneuvering tasks. Bøhn et al. [18] achieved the attitude control on fixed-wing UAV using the deep reinforcement learning method of on-policy proximal policy optimization (PPO). Fei et al. [19] presented a deep reinforcement learning control strategy trained using off-policy deep deterministic policy gradient (DDPG) and achieved goal-directed maneuvering for flapping-wing MAVs. Other challenging fields from games to robotics have employed a variety of state-of-art RL algorithms [20,21,22,23,24,25]. Haarnoja et al. [26] developed the SAC algorithm embedded with an automatic gradient-based temperature tuning method, which could achieve better performance without hyperparameter tuning for various tasks compared with other on-policy and off-policy algorithms. Combining the wing kinematics-based flight control strategy with the deep reinforcement learning approach may allow for the control of this highly coupled and nonlinear flight system without previous resolution. Through multiple explorations, the DRL controller is likely to show advantages in the fast achievement of control policy for 6-DoF flight stabilization even under large perturbations with no requirements on the prescribed database and the parameter determination.

Here we propose a novel wing kinematics-based controller optimized using deep reinforcement learning (DRL) for bumblebee hovering stabilization under large perturbations. We establish a high-fidelity Open AI Gym [27] environment through coupling a CFD data-driven aerodynamic model and a 6-DoF flight dynamic model. The control policy with action space of 4 is optimized using the off-policy Soft Actor–Critic (SAC) algorithm with automating entropy adjustment. The benchmark tests are conducted to investigate the feasibility of a wing kinematics-based DRL control strategy to achieve fast stabilization under full 6-DoF large disturbances for bumblebee hovering. Further analysis on the control performances demonstrates the superiority of the deep reinforcement learning strategy compared to the traditional linear strategies, which provides an efficient autonomous controller design for bioinspired flapping-wing micro air vehicles.

## 2. Materials and Methods

### 2.1. Morphological and Kinematic Bumblebee Models

**Figure 1:**
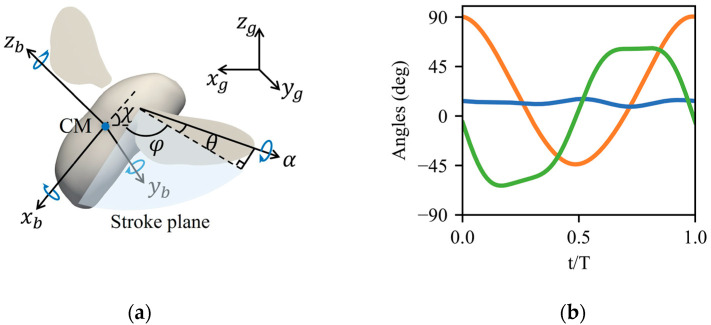
Morphological and kinematic models of bumblebee (*Bombus terrestris*): (**a**) Schematic of kinematic parameters defined in a global $\left(x_{g},\;y_{g},z_{g}\right)$ and a body-fixed $\left(x_{b},\;y_{b},z_{b}\right)$ coordinate systems. The roll angle *ρ*, pitch angle *χ*, and yaw angle *ψ* of the insect’s body are determined along the body axis of $x_{b}$, $y_{b},$ and $z_{b}$, respectively; (**b**) Wing kinematics of bumblebees in hovering flight are based on the experimental observations from Kolomenskiy et al. [28], where the positional angle φ (red), elevation angle θ (blue), and feathering angle α (green) are expressed in a Fourier series.

A wing-body model of the bumblebee (*Bombus terrestris*) is depicted in Figure 1a, whose body mass $m_{b}$ is 391 mg, body length *L* is 21 mm, wing mass $m_{w}$ is 0.76 mg, wing length *R* is 15.2 mm, and mean chord length $c_{m}$ is 4.1 mm. The kinematic model of a hovering bumblebee is built based on the experimental observations of Kolomenskiy et al. [28], which is defined by the three angles expressed as the first three terms of a Fourier series with respect to the stroke plane (Figure 1b): the positional angle φ, the elevation angle θ, and the feathering angle α. The positional angle is the rotation axis projection of the sweep angle within the stroke plane, the feathering angle is the geometric angle of attack around the rotation axis, the deviation angle between the stroke plane and rotation axis is the elevation angle. The wing beat frequency *f* for bumblebee hovering flight is 136 Hz, and the initial stroke amplitude *Φ* is 139.36°. The stroke plane angle *β* is 0° with the initial body angle *χ* equaling 45° for the hovering flight of bumblebees. For the rigid moving body, it is determined as three roll *ρ*, pitch *χ*, and yaw *ψ* body angles, in which the roll angle *ρ* is the rotational angle along body axis of $x_{b}$, the pitch angle *χ* is defined as the body inclination angle with respect to the horizontal plane, and the yaw angle represents the rotational angle along body axis of $z_{b}$.

### 2.2. Aerodynamic and Flight Dynamic Models for Bumblebee Hovering Flight

We construct a control environment in the framework of Open AI Gym [27] to achieve realistic the hovering flight of a bumblebee and provide fast response during the learning process. A CFD data-driven aerodynamic model (CDAM) by Cai et al. [11] is employed for fast prediction on the aerodynamic forces and torques, combined with a flight dynamic model based on Cai and Liu [13] which can mimic motions under large perturbations. The CDAM consists of a CFD-informed quasisteady model based on the blade element method for flapping wings and a simplified quasisteady approximation-based aerodynamic model for a moving body [11], which is a better alternative to the time-consuming CFD simulations. The flight dynamic model of a bumblebee applicable to large deviations is built by deriving the full dynamic equations extended from Gebert et al. [29] and Sun et al. [30]. The flight dynamic model is able to mimic the bumblebee wing-body interactions, where the wing kinematics are served as inputs and the insect’s motion can be solved in a fast and precise manner. The dynamic equations of the moving body are determined as

(1) mb+2mwA1oB1wRB1wLA2vR+A2vLIbd+A2oR+A2oLB2wRB2wL ddt vbcgωbbdωR0bωL0b=Fbbd+FbR+FbL+mbg−mbωbbd×vbcg−a1−b1Mbbd+MbR+MbL−ωbbd×Ibdωbbd−a2R+a2L−b2R+b2L,

where $m_{b}$, $m_{w}$ are the body and wing mass; $I_{bd}$ is a 3 × 3 matrix of the body moment of inertia ($I_{b,\;xx}=2.2\times 10^{-9}\;{\mathrm{kg}\;m}^{2}$, $I_{b,\;yy}=7.5\times 10^{-9}\;{\mathrm{kg}\;m}^{2}$, $I_{b,\;zz}=7.7\times 10^{-9}{\mathrm{kg}\;m}^{2}$); and Fbbd, FbR, FbL, Mbbd, MbR, and MbL calculated via CDAM denote the aerodynamic forces and torques on body and two wings. vbcg represents the velocity of the body’s center of mass, ωbbd denotes the angular velocity of the body, and $\omega_{R0b}$, $\omega_{L0b}$ represent the angular velocities of the right and left wings. The coefficients $A_{2oR}$ and $A_{2oL}$ can be expressed as $A_{2oR}=-m_{w}{\left[R_{hR}+R_{wgR}\right]}_{\times }{\left[R_{hR}\right]}_{\times }-m_{w}{\left[R_{hR}\right]}_{\times }{\left[R_{wgR}\right]}_{\times }+E_{wR2b}I_{w}E_{b2wR}$,$A_{2oL}=-m_{w}{\left[R_{hL}+R_{wgL}\right]}_{\times }{\left[R_{hL}\right]}_{\times }-m_{w}{\left[R_{hL}\right]}_{\times }{\left[R_{wgL}\right]}_{\times }+E_{wL2b}I_{w}E_{b2wL}$, where $I_{w}$ is the wing moment of inertia; $R_{hR}$, $R_{hL}$ denote the vector from the body center of mass to the wing base; $R_{wgR}$, $R_{wgL}$ denote the vector from the wing base to the wing center of mass; and $E_{wR2b}$, $E_{wL2b}$, $E_{b2wR}$, and $E_{b2wL}$ are the coordinate transformation matrix between the wing-fixed frame and the body-fixed frame. Detailed expressions of other coefficients $A_{1o}$, $B_{1wR}$, $B_{1wL}$,$\;A_{2vR}$, $A_{2vL}$, $B_{2wR}$, $B_{2wL}$, $a_{1}$, $b_{1}$, $a_{2R}$, $a_{2L}$, and $b_{2R}$, $b_{2L}$ are listed in Cai and Liu [13]. The flapping-wing dynamic equations are written as

(2) A2vR+CvRA2oR−CoRB2wR−CwR0A2vL+CvLA2oL−CoL0B2wL−CwL ddt vbcgωbbdωR0bωL0b−Mb2RMb2L= MbR−a2R−b2R+cRMbL−a2L−b2L+cL,

where $M_{b2R}$, $M_{b2L}$ denote the torques between the thorax of body and the right or left wing. Detailed expressions of the coefficients $C_{vR}$, $C_{vL}$, $C_{oR}$, $C_{oL}$, $C_{wR}$, $C_{wL}$ and $c_{R}$, $c_{L}$ are listed in Cai and Liu [13]. We further apply two equations by adding the wing kinematics-based control inputs, where $E_{dEulerR2sp}^{}$, $E_{dEulerL2sp}^{}$ are the coordinate transformation matrix that transfer the time derivative of wing Euler angles to the stroke plane frame; $E_{spR2b}^{}$, $E_{spL2b}^{}$ are the coordinate transformation matrix converting a vector from the stroke plane frame to the body-fixed frame, such as

(3) $${\dot{E}}_{dEulerR2sp}^{-1}E_{spR2b}^{’}\omega_{R0b}+E_{dEulerR2sp}^{-1}E_{spR2b}^{’}\frac{d\omega_{R0b}}{dt}=\left(\begin{array}{c} {\ddot{\varphi }}_{R}\\ {\ddot{\theta }}_{R}\\ {\ddot{\alpha }}_{R} \end{array}\right),$$

(4) $${\dot{E}}_{dEulerL2sp}^{-1}E_{spL2b}^{’}\omega_{L0b}+E_{dEulerL2sp}^{-1}E_{spL2b}^{’}\frac{d\omega_{L0b}}{dt}=\left(\begin{array}{c} {\ddot{\varphi }}_{L}\\ {\ddot{\theta }}_{L}\\ {\ddot{\alpha }}_{L} \end{array}\right).$$

By integrating Equations (1)–(4), the bumblebee motion could be solved using three inputs of wing kinematics φ,θ, α. Detailed expressions of all the coefficients in dynamic Equations (1)–(4) for the body and two wings can be found in Cai and Liu [13].

### 2.3. Wing Kinematics-Based Controller Design

**Figure 2:**
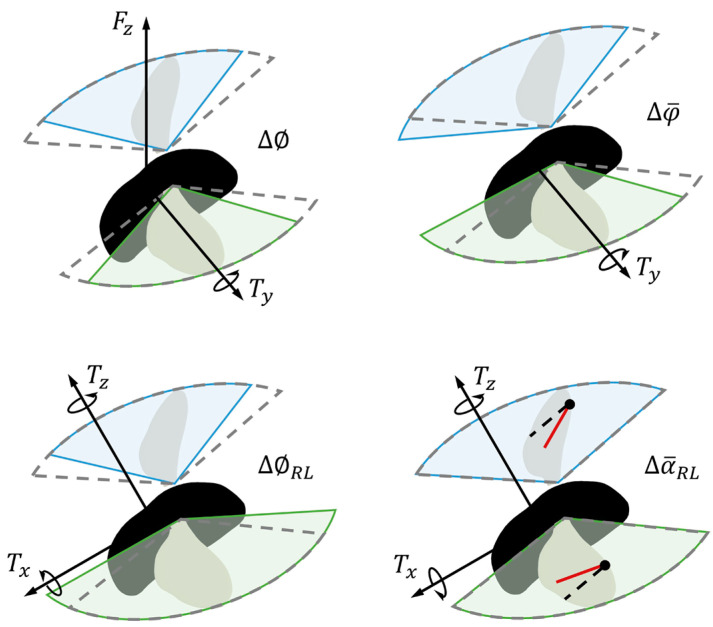
Aerodynamic forces and torques induced through wing kinematics variations: symmetric stroke amplitude variation ∆∅; symmetric mean positional angle variation $∆\bar{\varphi }$; asymmetric stroke amplitude variation between right and left wings $∆\emptyset_{RL}$; and asymmetric mean feathering angle variation between right and left wings $∆{\bar{\alpha }}_{RL}$. Dotted region: initial wing motion for trimmed hovering flight; shaded region with solid line: manipulated wing kinematics.

Cai and Liu [13] proposed a 6-DoF proportional derivative (PD) control strategy through directly tuning four wing kinematics parameters for bumblebee flight stabilization, leaving the *x* and *y* positions controlled indirectly by modifying the pitch and roll angles. Based on this successful trial, our controller design also selects four typical wing kinematics parameters to be served as the action space for deep reinforcement learning, and the aerodynamic forces and torques induced through wing kinematics variations are depicted in Figure 2: symmetric stroke amplitude variation ∆∅ will cause pitch torque $T_{y}$ and vertical forces $F_{z}$; symmetric mean positional angle variation $∆\bar{\varphi }$ may generate pitch torque $T_{y}$; and asymmetric stroke amplitude variation $∆\emptyset_{RL}$ and asymmetric mean feathering angle variation $∆{\bar{\alpha }}_{RL}$ between right and left wings could induce yaw $T_{z}$ and roll torques $T_{x}$.

Here, we propose a deep reinforcement learning (DRL) policy for insect-inspired flight control systems with the intention of achieving the bumblebee hovering stabilization under large perturbations. The bumblebee behaviors served as the Markov decision process (MDP) in continuous control. We build a state space with a dimension of 12 to observe the angular position, angular velocity, position, and velocity of the insect,

(5) $$s_{t}={\left[\psi ,\;\chi ,\;\rho ,\;\dot{\psi },\;\dot{\chi },\;\dot{\rho },\;x,\;y,\;z,\;\dot{x},\;\dot{y},\;\dot{z}\right]}^{T},$$

and an action space with a dimension of 4 to provide a continuous manipulation on the wing kinematics of a bumblebee,

(6) $$a_{t}={\left[∆\emptyset ,\;\;∆\bar{\varphi },\;\;∆\emptyset_{RL},\;\;∆{\bar{\alpha }}_{RL}\right]}^{T}.$$

**Figure 3:**
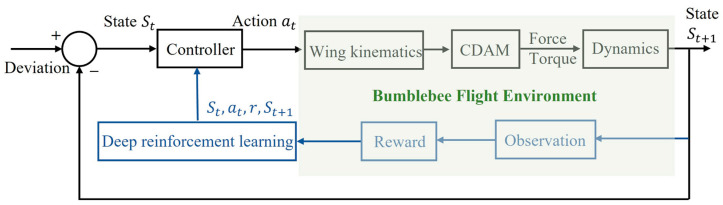
Schematic diagram of the wing kinematics-based bumblebee flight control system, where deep reinforcement learning gives solutions for controller design.

Figure 3 illustrates the schematic diagram of the wing kinematics-based bumblebee flight control system, where deep reinforcement learning gives solutions for controller design. The state transition for generating $s_{t+1}$ can be achieved through our bumblebee environment based on the closed-loop flight dynamic model with a feedback controller.

Since our flight control system requires continuous manipulation and updated strategy at the beginning of each wing-beat stroke, we choose the popular off-policy actor–critic algorithm based on the maximum entropy RL framework, Soft Actor–Critic (SAC) to train the policy [26]. There are three key components in the SAC algorithm: separate policy and value function-based actor–critic networks, high-efficiency data-reusing off-policy formulation, as well as stability and exploration-encouraging entropy maximization. The state value function is written as

(7) $$V\left(s_{t}\right)=E_{a_{t}\sim \pi }\left[Q\left(s_{t},a_{t}\right)-\alpha \log\pi \left(a_{t}|s_{t}\right)\right].$$

Thus, the Q value function based on soft Bellman equation [25,26] is given by

(8) $$Q\left(s_{t},a_{t}\right)=r\left(s_{t},a_{t}\right)+\gamma E_{s_{t+1},\;\;a_{t+1}}\left[Q\left(s_{t+1},a_{t+1}\right)-\alpha \log\pi \left(a_{t+1}|s_{t+1}\right)\right],$$

where r is the one-step reward, E denotes the mathematical expectation, γ is the discount factor, and π is the adopted policy. Here, α controls how important the entropy term is, known as the temperature parameter. The SAC updates the policy to minimize the Kullback–Leibler (KL) divergence [25,26],

(9) πnew=arg minπ′∈ΠDKLπ′·|st∥exp1αQπoldst,·Zπoldst,

where Π denotes the family of Gaussian distributions and Z represents the partition function for distribution normalization. The parameters of the soft Q-function θ are trained by [25,26],

(10) $$J_{Q}\left(\theta \right)=E_{\left(s_{t},\;\;a_{t}\right)\sim D}\left[\;\frac{1}{2}{\left(Q_{\theta }\left(s_{t},a_{t}\right)-\left(r\left(s_{t},a_{t}\right)+\gamma E_{s_{t+1},\;\;a_{t+1}}\left[V_{\bar{\theta }}\left(s_{t+1}\right)\right]\right)\right)}^{2}\right],$$

where D is the replay buffer storing the transitions $\left[s_{t},a_{t},\;r,s_{t+1}\right]$. A soft update is performed in target value network,

(11) $$\bar{\theta }\leftarrow \tau \theta +\left(1-\tau \right)\bar{\theta },$$

where τ denotes the step factor and $\bar{\theta }$ is an exponentially moving average of the weights. The policy network with parameter ϕ is updated by [25,26],

(12) $$J_{\pi }\left(\phi \right)=\nabla_{\theta }D_{KL}\left(\pi_{\phi }\left(\cdot |s_{t+1}\right)∥\exp\left(\frac{1}{\alpha }Q_{\theta }\left(s_{t},\cdot \right)-\log Z_{\theta }\left(s_{t}\right)\right)\right)=E_{a_{t}\sim \pi }\left[\;\log\pi_{\phi }\left(a_{t}|s_{t}\right)-Q_{\theta }\left(s_{t},a_{t}\right)+\log Z_{\theta }\left(s_{t}\right)\right].$$

Since a suboptimal temperature may cause poor performance in maximum entropy RL [25], a constrained formulation for automatically tuning the temperature hyperparameter has been employed in SAC without the requirement for hyperparameter tuning in every task. The optimal temperature parameter α in every step can be learned by minimizing the same objective function [25,26],

(13) $$J\left(\alpha \right)=E_{a_{t}\sim \pi_{t}}\left[-\alpha \log\pi_{t}\left(a_{t}|s_{t}\right)-\alpha H_{0}\right],$$

where $H_{0}$ is the desired minimum expected entropy. The Soft Actor–Critic (SAC) with automating entropy adjustment has been evaluated through a variety of benchmark and real-world tasks of robotics [26], which could achieve outstanding asymptotic performance and sample efficiency compared with other off-policy and on-policy algorithms [20,21,22,23,24].

## 3. Results

### 3.1. Deep Reinforcement Learning Policy

The goal of the bumblebee flight control system is to restore the angular position and position to the initial equilibrium state after large angular velocity or velocity perturbations via several strokes controlling. The reward design is determined as a negative cost function composed of stability cost and control cost, such as

(14) $$Reward=-\left(\lambda_{p}e_{p}{}^{2}+\lambda_{v}e_{v}{}^{2}+\lambda_{R}e_{R}{}^{2}+\lambda_{\omega }e_{\omega }{}^{2}+\lambda_{a}a_{t}{}^{2}+\lambda_{a}{\dot{a_{t}}}^{2}\right).\;$$

The stability cost is defined as the errors between current states and target states, where $e_{p}$ denotes the position errors of ∆x, ∆y, and ∆z; $e_{v}$ denotes the velocity errors of $∆\dot{x},\;∆\dot{y},\;\mathrm{and}\;∆\dot{z}$; $e_{R}$ denotes the attitude errors of ∆ψ, ∆χ, and ∆ρ; and $e_{\omega }$ denotes the angular velocity errors of $∆\dot{\psi },\;∆\dot{\chi },\;\mathrm{and}\;∆\dot{\rho }$. The action cost $a_{t}$ and action changing rate $\dot{a_{t}}$ are also included in reward design as the control cost to ensure the stable wing kinematics and equilibrium state in trimmed hovering flight of bumblebee. Note that all the quantities of time, length, velocity, mass, force, and torque in our simulation environment have been processed and expressed as a dimensionless form, which leaves the bound value of the six reward terms with quite different orders of magnitudes ranging over *O* ($10^{0}$)–*O* ($10^{4}$). To ensure the relatively equivalent contribution for each reward component and minimize the attitude, position, velocity, and control errors at the same time, we design the scaling parameters as

(15) $$\lambda_{p}:\lambda_{v}:\lambda_{R}:\lambda_{\omega }:\lambda_{a}:\lambda_{a}=10^{0}:10^{4}:10^{0}:10^{4}:10^{0}:10^{0},\;$$

To balance and scale the differences in orders of magnitudes of these nondimensional values. Through a variety of training verification, we found that further precise adjustment for each parameter may not enhance the training performance largely, which demonstrates the current scaling-based parameters in the reward design are rational for learning achievement. The reinforcement learning of the SAC algorithm has advantages in the fast achievement of control policy via exploration with no requirements on the prescribed database and the precise determination of control parameters.

**Figure 4:**
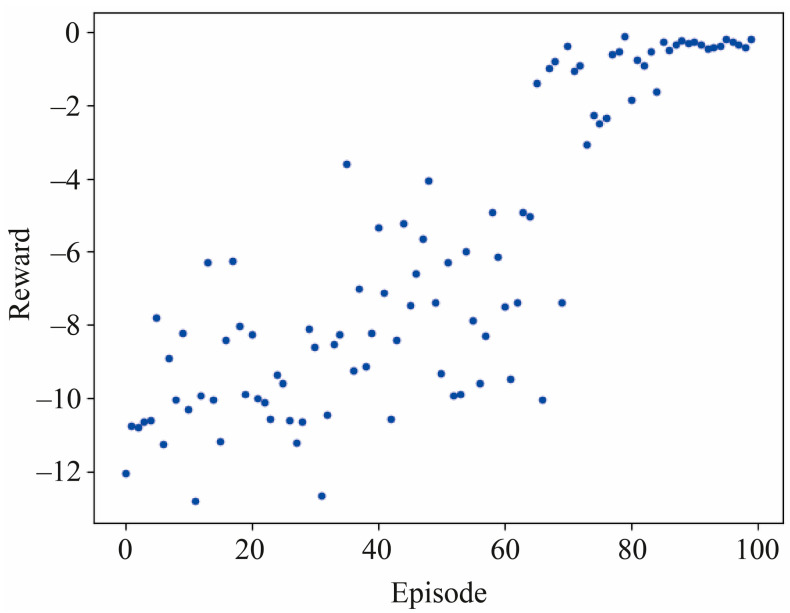
Training process illustrated by the learning curve with obtained reward at the end of each exploration episode.

Considering the realistic morphology and kinematics of insects, we set the limitations of the action space, such as the maximum rising in stroke amplitude for 20% or the maximum deviation in mean positional and feathering angle for 20° to avoid overlapping of two wings. We also modify the hyperparameters based on Haarnoja et al. [26] and utilize several tricks such as a reward scale incorporated with SAC to improve the training robustness. The training process illustrated by a learning curve with obtained reward at the end of each exploration episode is shown in Figure 4, where the reward is maximized via minimizing the error between the current state and the equilibrium state to be closer to zero. The training process showed in Figure 4 is quite similar as most of the successful DRL cases [25,26], in which the learning curve appears random and slowly increases at the beginning while it rises fast and even becomes stable during the last several episodes. Since the SAC algorithm could enhance the action selection randomness, and meanwhile encourage more exploration during the training process [31], the actor generates random actions based on the current policy during initial episodes, and the feedback of the environment will be stored into the experience replay buffer for updating the network at each flapping stroke. After sufficient explorations for dozens of episodes, the updated policy may provide actions to achieve better performance until the reward is optimized (the error tolerance is defined as $\left|r-0\right|<$0.5 considering 5% of the initial reward value). The accumulated negative reward could converge the highest, which is close to zero after randomly giving deviations at the beginning of each episode and exploring actions for 5000 steps (50 flapping strokes for each episode). The number of episodes that served as one of the initial hyperparameters was determined as 100 previously, which was demonstrated to be sufficient for achieving training performance.

### 3.2. Stabilization Control under Large Perturbations

**Figure 5:**
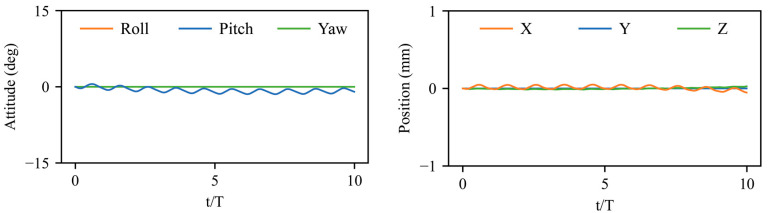
Trimmed state of a hovering bumblebee in equilibrium without perturbation.

The trimmed state of a hovering bumblebee is illustrated in Figure 5, which reaches a stable periodic state with initial trimmed wing kinematics and maintains equilibrium without perturbation for 10 strokes. A slight body oscillation is induced by the symmetric reciprocation motion of the two flapping wings (Figure 1), involving pitch motion, forward/backward motion, and vertical motion. The goal of flight control is to restore the attitude and position of the bumblebee after disturbances to ${\left[\rho_{0},\;\chi_{0},\;\psi_{0}\right]}^{T}={\left[0,\;45,\;0\right]}^{T}$(°) and ${\left[x_{0},\;y_{0},z_{0}\right]}^{T}={\left[0,\;0,\;0\right]}^{T}$(mm). In our control results, all the pitch angles have been illustrated as χ−45°.

**Figure 6:**
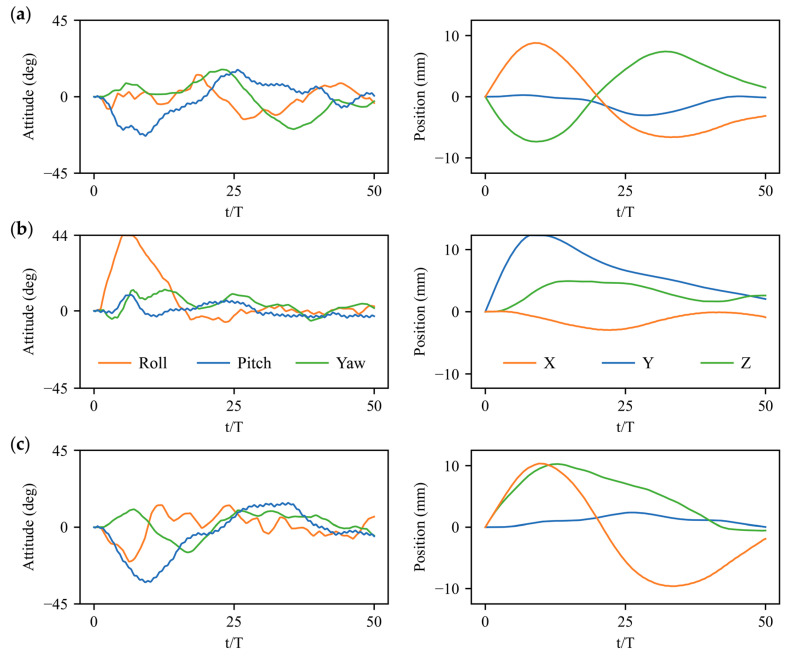
Attitude and position control results under velocity perturbations: (**a**) Horizontal perturbation in direction of $x_{g}$; (**b**) Lateral perturbation in direction of $y_{g}$; (**c**) Vertical perturbation in direction of $z_{g}$.

**Figure 7:**
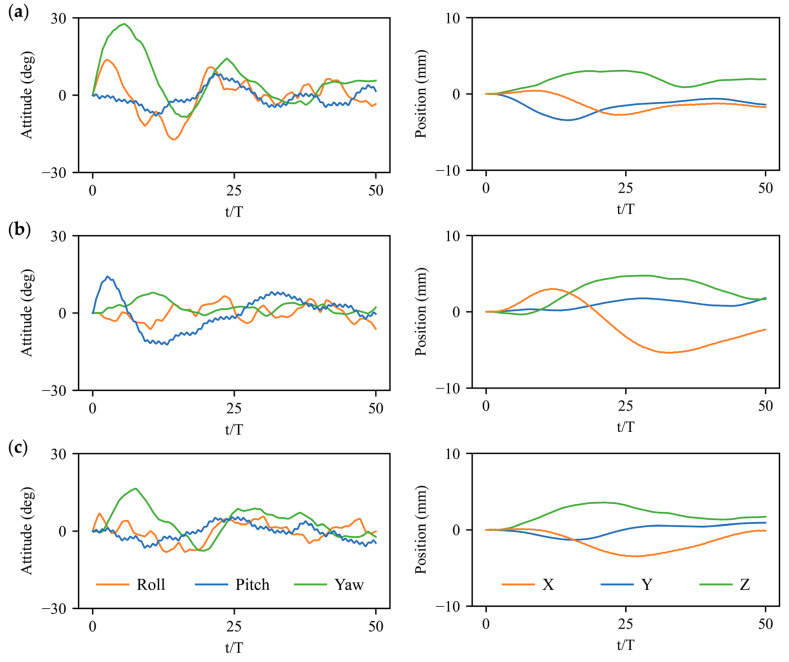
Attitude and position control results under angular velocity perturbations: (**a**) Roll perturbation along body axis of $x_{b}$; (**b**) Pitch perturbation along body axis of $y_{b}$; (**c**) Yaw perturbation along body axis of $z_{b}$.

Experiments on bumblebee flight control under large perturbations are conducted through applying large angular velocity perturbations along the body axis of $\left(x_{b},\;y_{b},z_{b}\right)$ and large velocity perturbations in directions of $\left(x_{g},\;y_{g},\mathrm{and}\;z_{g}\right)$, which mimics the impact of wind-gust disturbance on the insect’s body [2,32]. Even gentle air currents can cause large disruptions to the intended flight path [1] according to the perturbation experiments of bumblebees [13,32] and fruit flies [1,2]. We employ the trained deep reinforcement learning policy as control strategy after adding the angular velocity disturbances 3% $\omega_{ref}$ (≈20 rad/s) and the velocity disturbances 3% $U_{ref}$ (≈ 0.3 m/s) [13,32] to the trimmed hovering state of a bumblebee. Here, the reference angular velocity and the reference velocity are defined as the wingtip angular velocity and wingtip velocity of the bumblebee in hovering flight, such as $\omega_{ref}=2\emptyset f$ and $U_{ref}=2\emptyset fR$, where R denotes the wing length and ∅ and *f* are the stroke amplitude and flapping frequency. The flight system in equilibrium with initial trimmed wing kinematics is perturbed by the angular velocity and velocity disturbances at the first flapping stroke persisting for one stroke cycle. After a time delay of 1*T* latency, the actions (active wing kinematics manipulation) generated by the DRL policy will be added into the flight system. Figure 6 and Figure 7 depict the control results in terms of three body attitude (roll, pitch, and yaw angles) and three body positions (*X*, *Y*, and *Z*) under, three horizontal, lateral, and vertical velocity perturbations, as well as three roll, pitch, and yaw angular velocity perturbations, respectively. Although all the large perturbations in different directions result in deviations in rotational angles and body positions, the deep reinforcement learning (DRL) controller based on the action space of four wing kinematics can largely achieve the 6-DoF stabilization for bumblebee hovering flight even in underactuated condition.

**Figure 8:**
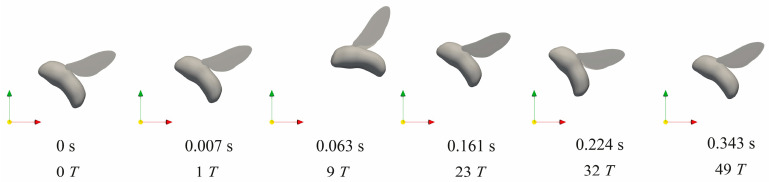
Flight sequence of a hovering bumblebee after vertical velocity perturbation.

The rotational control based on DRL policy can be achieved at around 20 wing-beat strokes, which is slightly slower than the experimental observations on various insect flights [1,2,3,9,10]. More restoring time of approximately 40–50 strokes is needed to obtain the translational control after large perturbations, which may be less essential compared with the attitude stabilization [33]. The gust rejection and flight profile after the disturbance can be visualized through the dynamic sequence of the bumblebee motion, for instance, the detailed control process with the variation of body rotation and movement after vertical velocity perturbation in the direction of $z_{g}$ in Figure 8. The hovering bumblebee in equilibrium (0 s) encounters a vertical velocity disturbance at the initial stroke cycle (~0.007 s) resulting in a rapid movement in the vertical direction. After one stroke time delay, it takes active wing kinematics manipulations for several flapping cycles, during which the bumblebee first pitches down 40° and restores the body attitude quickly within 0.161 s. The pitch response of the bumblebee induces the backward and forward motion of the body, which takes more stroke cycles to return to the initial position. Although the translational control requires relatively more restoring time due to the indirect adjustments from an action space of 4 under full 6-DoF disturbances, the underactuated DRL controller has great potential to simplify the actuator-based fabrication in flapping-wing MAVs.

The quantification analysis on the control results of the deep reinforcement learning (DRL) strategy and a comparison to the traditional PD control strategy are further provided. Here, two indices are introduced for the evaluation of control performance: the maximum attitude or position displacement $d_{max}$ from the equilibrium state and the correction time $t_{c}$ expressed in wing-beat cycles [2]. Through calculating the rotational and translational differences (‘errors’) between 0 and 50 wing beat cycles [2], 80% response curves of the attitude and position induced by 6-DoF disturbances can be effectively restored toward the stable state within 10% of the maximum displacement, which indicates the control capability of the deep reinforcement learning strategy. Table 1 and Table 2 show the detailed values of $d_{max}$ and $t_{c}$ based on the time evolutions of body attitudes and positions under horizontal, lateral, and vertical velocity perturbations using a current DRL controller and traditional PD controller [13]. The maximum displacements of roll, pitch, and yaw attitudes are comparable in the DRL and PD controls, in which the mean values turn out to be 28°±17° for the DRL controller and 26°±16° for the PD controller. However, lower displacements exist in the position control of X, Y, and Z with the DRL controller, whose mean $d_{max}$ shows a reduction of 40% compared with PD control results. Moreover, although the DRL controller requires slightly more correction time $t_{c}$ for translational deviations, it presents a significant advance in rotational stabilization with the time saving of 50% (19.5±3.5 wing beats) compared with the PD controller (37.3±9.7 wing beats). Better control performances in terms of displacement reduction and restoring time demonstrate the superiority of deep reinforcement learning compared to traditional linear strategies.

### 3.3. Physical Mechanisms of Control Strategy

**Figure 9:**
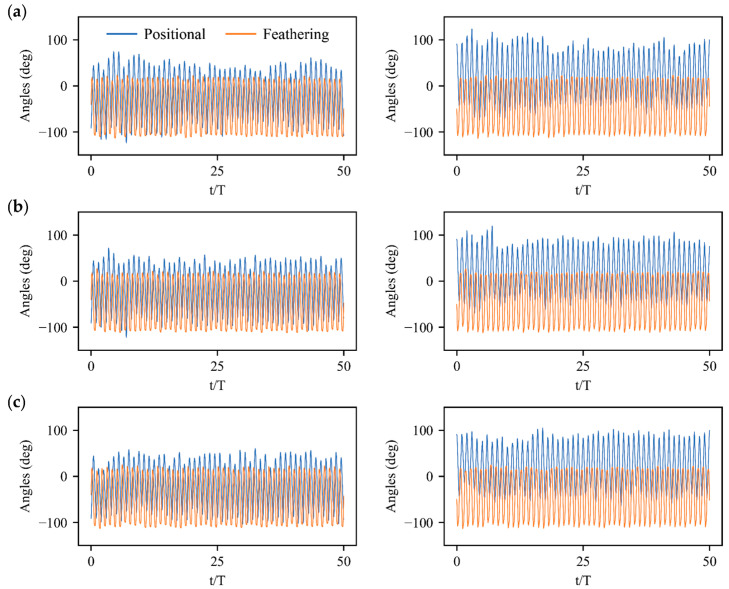
Wing kinematics manipulations of left and right wings under velocity perturbations: (**a**) Horizontal perturbation in direction of $x_{g}$; (**b**) Lateral perturbation in direction of $y_{g}$; (**c**) Vertical perturbation in direction of $z_{g}$.

**Figure 10:**
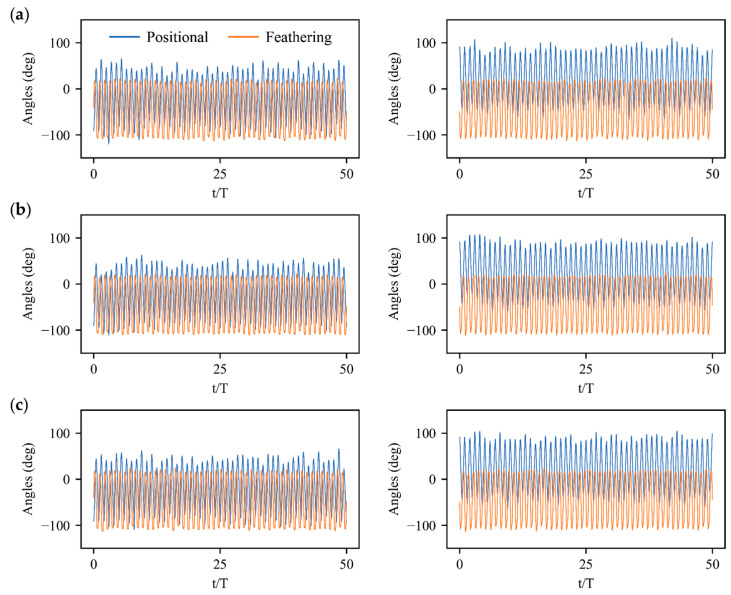
Wing kinematics manipulations of left and right wings under angular velocity perturbations: (**a**) Roll perturbation along body axis of $x_{b}$; (**b**) Pitch perturbation along body axis of $y_{b}$; (**c**) Yaw perturbation along body axis of $z_{b}$.

The control strategy with action inputs expressed as the wing kinematics manipulations of left and right wings have been shown in Figure 9 and Figure 10. Since the bumblebee normally activates its muscles once in one stroke cycle [34], the control policy applies actions to the wing kinematics at the beginning of each stroke cycle. A smooth step function is further employed to ensure the wing kinematics transition between successive strokes with a transition time of 0.1*T* [13]. The trained policy calls forth the commands with the symmetric and asymmetric variations in positional and feathering angles of two wings, which are highly correlated with the generation of aerodynamic forces and torques (Figure 2) resulting in the physical response of the flight system. For instance, the control strategies for velocity disturbances in $x_{g}$, $z_{g}$ directions and angular velocity disturbances along body axis of $y_{b}$ show significant symmetric variations in the amplitude and mean value of positional angle. The pitch torques $T_{y}$ largely generated by a symmetric mean positional angle as well as the vertical and horizontal forces $F_{z}$, $F_{x}$ mainly from symmetric stroke amplitude dominate the remarked pitch-up/down deviations and meanwhile induce the forward/backward and vertical motions. Similarly, significant asymmetric variations in the stroke amplitude and mean feathering angle of left and right wings dominate the control strategies for velocity disturbance in $y_{g}$ direction and angular velocity disturbances along the body axis of $x_{b}$, $z_{b}$. The remarkable rotational responses in roll and yaw directions with lateral movements appear due to the synchronous or opposite roll and yaw torques $T_{x}$, $T_{z}$ induced by the asymmetry in the stroke amplitude and mean feathering angle, as well as the lateral forces $F_{y}$ produced by the asymmetry in mean values of left- and right-wing feathering angles.

The flight system is highly coupled as the body’s natural modes of motion couple with the periodic aerodynamic and inertial forces associated with flapping wings [4]. Strong coupling between roll and yaw motions can be noticed in time evolutions of Figure 7a, and the lateral velocity perturbation in the direction of $y_{g}$ may also induce significant rotational deviations in roll angles (Figure 6b). The coupling phenomenon can be explained by the aerodynamic performance of the leading-edge vortex (LEV), where the side-translational velocity may cause the difference in relative velocities of left and right wings as well as the axial velocities of LEVs [35]. This will lead to an asymmetry in the aerodynamic lift production of the two wings, which further generates roll moment for body rotation. Moreover, due to the asymmetric moderations in the stroke amplitude and mean feathering angle of left and right wings, the significant synchronous or opposite roll and yaw torques $T_{x}$, $T_{z}$ as well as the moderate lateral forces $F_{y}$ are produced and force the coupling sideways motions of the insect’s body. Meanwhile, significant pitch deviation and vertical motion in the direction of $z_{g}$ can be caused via horizontal velocity perturbation in the direction of $x_{g}$ (Figure 6a). The coupling features can be explained by the variation in aerodynamic drags in both down- and up-strokes due to varied relative velocity, which causes a cycle-averaged horizontal force around the center of mass producing a pitch moment for body rotation [35]. Additionally, the pitch torques $T_{y}$ generated by symmetric variation in the mean positional angle as well as the vertical and horizontal forces $F_{z}$, $F_{x}$ produced via symmetric stroke amplitude manipulation can further dominate the coupling longitudinal responses in terms of the forward/backward and vertical motions as well as the pitch-up/down deviations.

The previous studies on linear control strategy employed eigenvalue and eigenvector analyses to decouple and resolve the longitudinal and lateral equations [6,7,14,15]. However, the sideways motions also have an impact on the longitudinal motions, as large angular perturbations along roll and yaw axis may induce remarked deviations in pitch angles as well as forward/backward and vertical motions according to the responses showed in Figure 6 and Figure 7. The linear assumption may not be feasible as the nonlinearity still exists in the correlations between the wing kinematics modulation and the production of aerodynamic forces and torques. Thus, the DRL controller enables flight control in highly-coupled and nonlinear systems without previous resolution. More importantly, the determination of precise control parameters via the Laplace transformation and root locus approach [11,13] in traditional linear strategies are not necessary for the DRL control strategy, which has proved to be of great potential in fast policy achievement without precise treatments for control parameter implementation. Therefore, the 6-DoF four-wing kinematics-based DRL control strategy will further simplify the actuator-based fabrication and inspire the autonomous controller design for insect-inspired flapping-wing MAVs.

## 4. Conclusions

In this study, we have developed an integrated simulation framework with a bio-inspired flight intelligence controller optimized by deep reinforcement learning (DRL) tasked with achieving bumblebee hovering stabilization under large perturbations. A high-fidelity Open AI Gym environment is established coupling a CFD data-driven aerodynamic model and a 6-DoF flight dynamic model tailored to provide fast aerodynamics prediction and mimic different flight conditions. We propose a unique wing kinematics-based flight control strategy optimized using the Soft Actor–Critic (SAC) algorithm, which is proven to be successful in underactuated condition with an action space of 4 for stabilization under full disturbances from 6 DoF. Fast control after large perturbations could be obtained in body attitude stabilization of yaw, pitch, and roll angles while it takes more wing-beat cycles for body position stabilization of horizontal, lateral, and vertical motions. Better control performances in terms of displacement reduction and restoring time demonstrate the superiority of deep reinforcement learning compared to traditional linear strategies. The DRL controller enables flight control in highly coupled and nonlinear systems without previous resolution, and has great potential in fast control policy achievement without precise treatments for control parameter implementation. This 6-DoF wing kinematics-based DRL control strategy may provide an efficient autonomous controller design for bioinspired flapping-wing micro air vehicles.

## Figures and Tables

**Table 1:** The maximum attitude or position displacements $d_{max}$ from the equilibrium state under horizontal, lateral, and vertical velocity perturbations using a proportional derivative (PD) controller and deep reinforcement learning (DRL) controller.

	Horizontal			Lateral			Vertical		
$d_{max}$	X (mm)	Pitch (°)	Z (mm)	Roll (°)	Y (mm)	Yaw (°)	X (mm)	Pitch (°)	Z (mm)
PD	16	11	0	23	18	28	15	42	31
DRL	9	23	7	45	13	12	10	32	10

**Table 2:** The correction time $t_{c}$ expressed in wing-beat cycles under horizontal, lateral, and vertical velocity perturbations using a proportional derivative (PD) controller and deep reinforcement learning (DRL) controller.

	Horizontal			Lateral			Vertical		
$t_{c}$	X (*T*)	Pitch (*T*)	Z (*T*)	Roll (*T*)	Y (*T*)	Yaw (*T*)	X (*T*)	Pitch (*T*)	Z (*T*)
PD	50	35	0	31	44	35	19	47	61
DRL	20	20	50	16	50	19	42	23	50

## Data Availability

The data generated and/or analyzed as well as the sources code during the current study are not publicly available due to their use in an undergoing project but could be available from the corresponding author on reasonable request.
